# Protocol for a clinical trial of text messaging in addition to standard care versus standard care alone in prevention of type 2 diabetes through lifestyle modification in India and the UK

**DOI:** 10.1186/s12902-018-0293-8

**Published:** 2018-09-10

**Authors:** Hazel Thomson, Nick Oliver, Ian F. Godsland, Ara Darzi, Weerachai Srivanichakorn, Azeem Majeed, Desmond G. Johnston, Arun Nanditha, Chamukuttan Snehalatha, Arun Raghavan, Priscilla Susairaj, Mary Simon, Krishnamoorthy Satheesh, Ambady Ramachandran, Stephen Sharp, Kate Westgate, Søren Brage, Nick Wareham

**Affiliations:** 1 Diabetes and Endocrinology, Imperial College London St Mary’s Hospital Campus, Norfolk Place, London, W2 1PG UK; 2 Department of Surgery and Cancer, Imperial College London, St Mary’s Hospital Campus, Norfolk Place, London, W2 1PG UK; 3 Department of Medicine, Faculty of Medicine Siriraj Hospital, Mahidol University, 2 Wang Lang Road, Bangkok Noi, Bangkok, 10700 Thailand; 4 Primary Care and Public Health, Imperial College London Charing Cross Hospital Campus, Reynolds Building, Hammersmith, London, w6 8RP UK; 5 India Diabetes Research Foundation and Dr. A. Ramachandran’s Diabetes Hospitals, Chennai, 28 Marshalls Road, Egmore, Chennai, 600 008 India; 6 MRC Epidemiology Unit, University of Cambridge School of Clinical Medicine, Institute of Metabolic Science, Cambridge Biomedical Campus, Cambridge, CB2 0SL UK

**Keywords:** Short messaging service, Prediabetes, HbA1c, Diabetes prevention, Randomised controlled trial

## Abstract

**Background:**

Type 2 diabetes is a serious clinical problem in both India and the UK. Adoption of a healthy lifestyle through dietary and physical activity modification can help prevent type 2 diabetes. However, implementing lifestyle modification programmes to high risk groups is expensive and alternative cheaper methods are needed. We are using a short messaging service (SMS) programme in our study as a tool to provide healthy lifestyle advice and an aid to motivation. The aim of the study is to assess the efficacy and user acceptability of text messaging employed in this way for people with pre-diabetes (HbA1c 6.0% to ≤6.4%; 42–47 mmol/mol) in the UK and India.

**Methods/design:**

This is a randomised, controlled trial with participants followed up for 2 years. After being screened and receiving a structured education programme for prediabetes, participants are randomised to a control or intervention group. In the intervention group, text messages are delivered 2–3 times weekly and contain educational, motivational and supportive content on diet, physical activity, lifestyle and smoking. The control group undergoes monitoring only. In India, the trial involves 5 visits after screening (0, 6, 12, 18 and 24 months). In the UK there are 4 visits after screening (0, 6, 12 and 24 months). Questionnaires (EQ-5D, RPAQ, Transtheoretical Model of Behavioural Change, and food frequency (UK)/24 h dietary recall (India)) and physical activity monitors (Actigraph GT3X+ accelerometers) are assessed at baseline and all follow-up visits. The SMS acceptability questionnaires are evaluated in all follow-up visits. The primary outcome is progression to type 2 diabetes as defined by an HbA1c of 6.5% or over(India) and by any WHO criterion(UK). Secondary outcomes are the changes in body weight, body mass index, waist circumference, blood pressure, fasting plasma glucose; lipids; proportion of participants achieving HbA1c ≤6.0%; HOMA-IR; HOMA-β; acceptability of SMS; dietary parameters; physical activity and quality of life.

**Discussion:**

The study is designed to assess the efficacy of tailored text messaging in addition to standard lifestyle advice to reduce the progression from prediabetes to type 2 diabetes in the two different countries.

**Trial registration:**

ClinicalTrials.gov; NCT01570946, 4^th^ April 2012 (India); NCT01795833, 21^st^ February 2013 (UK).

## Background

The burden of type 2 diabetes in both India and the UK is high and predicted to rise. In India, the prevalence in adults is 7.3%, although figures vary from state to state and in urban versus rural settings [1–3]. In England, 6.7% of the population aged over 17 years are known to have diabetes [4]. A large proportion of both populations have intermediate levels of hyperglycaemia, or so-called pre-diabetes, and this constitutes an identifiable sub-group who are at high risk of progression to diabetes [5–7] and in whom preventive interventions have been shown to be effective [8–12].

A major challenge in applying high risk individual-level diabetes prevention strategies to large sub-groups within the population is that the successful lifestyle modification interventions employed in clinical trials are labour intensive and expensive and thus difficult to operationalise at scale. Our intervention strategy attempts to overcome this issue of scalability by employing a lifestyle modification programme delivered by short messaging service (SMS) in both India and the UK. Text messaging is cheap and feasible and has been successfully used to modify behaviour in other contexts such as smoking cessation [13, 14].

The primary outcome is progression to diabetes. Secondary outcomes include cardiovascular disease (CVD) risk factors, dietary intake, physical activity, and quality of life (EQ-5D).

## Methods/design

This is a randomised, controlled trial with participants followed up for 2 years.

In *India*, pre-screening occurs in the workplace (Indian railways and other employers) and is performed by dedicated trained research staff [15]. Men and women are selected for subsequent screening if, on a questionnaire and a short anthropometric assessment, they have 3 or more of the following diabetes risk factors: age 35 to 55 years; family history of diabetes; personal history of hypertension; sedentary life-style; previous gestational diabetes; known prediabetes; waist circumference ≥ 90 cm in males, ≥80 cm in females; body mass index (BMI) ≥ 23 kg/m^2^. Screening is based on haemoglobin A1c (HbA1c) levels, measured using a point-of-care device using capillary blood. People are invited to join the trial if they have HbA1c levels in the upper prediabetes range (HbA1c 6.0% to ≤6.4%, 42 to 47 mmol/mol) [16–18]. Pre-screening and screening are typically performed on the same day. People who are discovered to have diabetes are referred for routine care by their physicians.

**Fig. 1:**
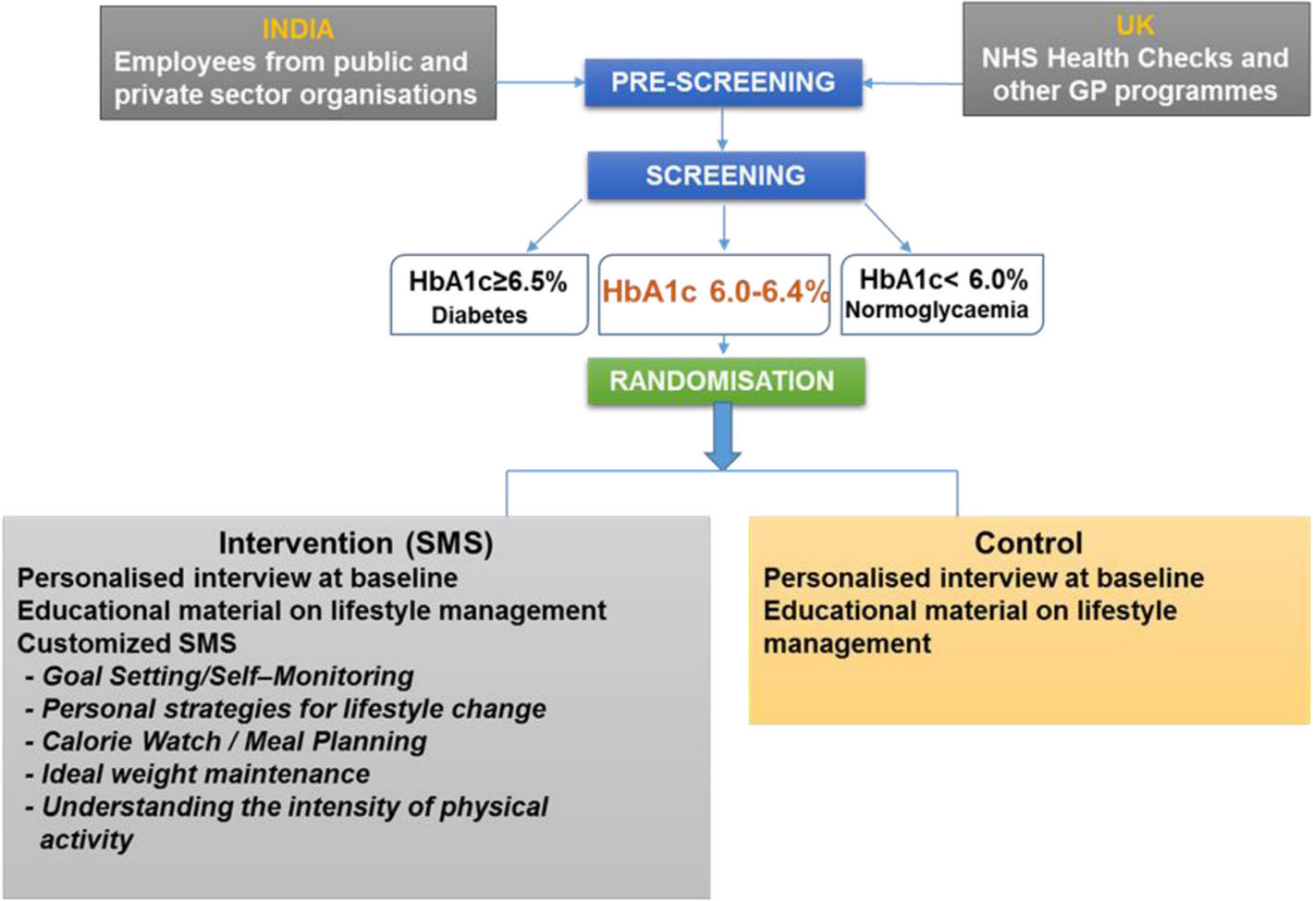
Recruitment flowchart in India and the UK

In the *UK*, pre-screening identifies people as having prediabetes of any degree (by national and international criteria for either glucose or HbA1c) [19] in the National Health Service (NHS) Health Check programme, or in other in-house health screening programmes in primary care. The NHS Health Check is offered to men and women aged 40 to 74 years. In-house programmes may be offered to people of 18 years or over. Following consent, participants have their HbA1c measured and are considered eligible for the trial if the result is in the upper prediabetes range [17, 18] (as in India). If HbA1c is measured in the pre-screening stage, and if this is within 1 week of full screening, then this value is accepted for inclusion in the trial. Anyone whose HbA1c is in the diabetic range is referred for routine care for their diabetes. The study has been adopted by the National Institute for Health Research Clinical Research Network (NIHR CRN) and trained CRN staff perform much of the recruitment and subsequent activity. Recruitment flowchart in India and the UK is shown in Fig. 1.

In *India*, the trial involves 5 visits after screening (0, 6, 12, 18 and 24 months). In the *UK* there are 4 visits after screening (0, 6, 12 and 24 months).

### Screening visit

In India, participants have their height, weight, waist circumference, blood pressure and heart rate measured. A point of care device (Bio-Rad, boronate affinity chromatography) is used for HbA1c testing. Random glucose (Accu-Check, Roche Diagnostics) is also measured for all participants.

In the UK, participants attend fasting and their height, weight, waist circumference, hip circumference, blood pressure and heart rate are measured. Venous blood (15 ml) is taken for measurement of HbA1c, glucose and fasting lipids. A serum sample is stored for biomarker measurement. A random urine sample is taken for measurement of the albumin: creatinine ratio.

In both countries, questionnaires are completed (EQ-5D, RPAQ, Transtheoretical Model of Behavioural Change (TTM), and food frequency/24 h dietary recall). People are classified by TTM stage. Physical activity monitors (Actigraph GT3X+ triaxial accelerometers) are fitted and participants are asked to wear them on the lower back for 7 days and nights. Pre-paid addressed envelopes are provided for the return of monitors.

### Education and randomization visit

In India, participants attend fasting. Venous blood (15 ml) is taken for measurement of fasting glucose and lipids. A serum sample is stored for biomarker measurement. In both India and the UK, a structured education programme for prediabetes is delivered including: definition; implications for progression to diabetes; prevention of type 2 diabetes; healthy lifestyle advice including dietary and physical activity advice, supplemented by written materials.

Participants are then randomised to a control (standard care) or intervention (standard care plus SMS) group.

### Follow-up visits

These are the 6, 12, 18 and 24 month follow-up visits. Participants are fasting for all visits in the UK, and only for the 12- and 24-month visits in India. The 18-month visit is omitted in the UK. The assessments and measurements are as per baseline;

- TTM staging is decided and SMS content modified on this basis. The acceptability of receiving the text messages is assessed by questionnaire at each follow-up visit.

**Fig. 2:**
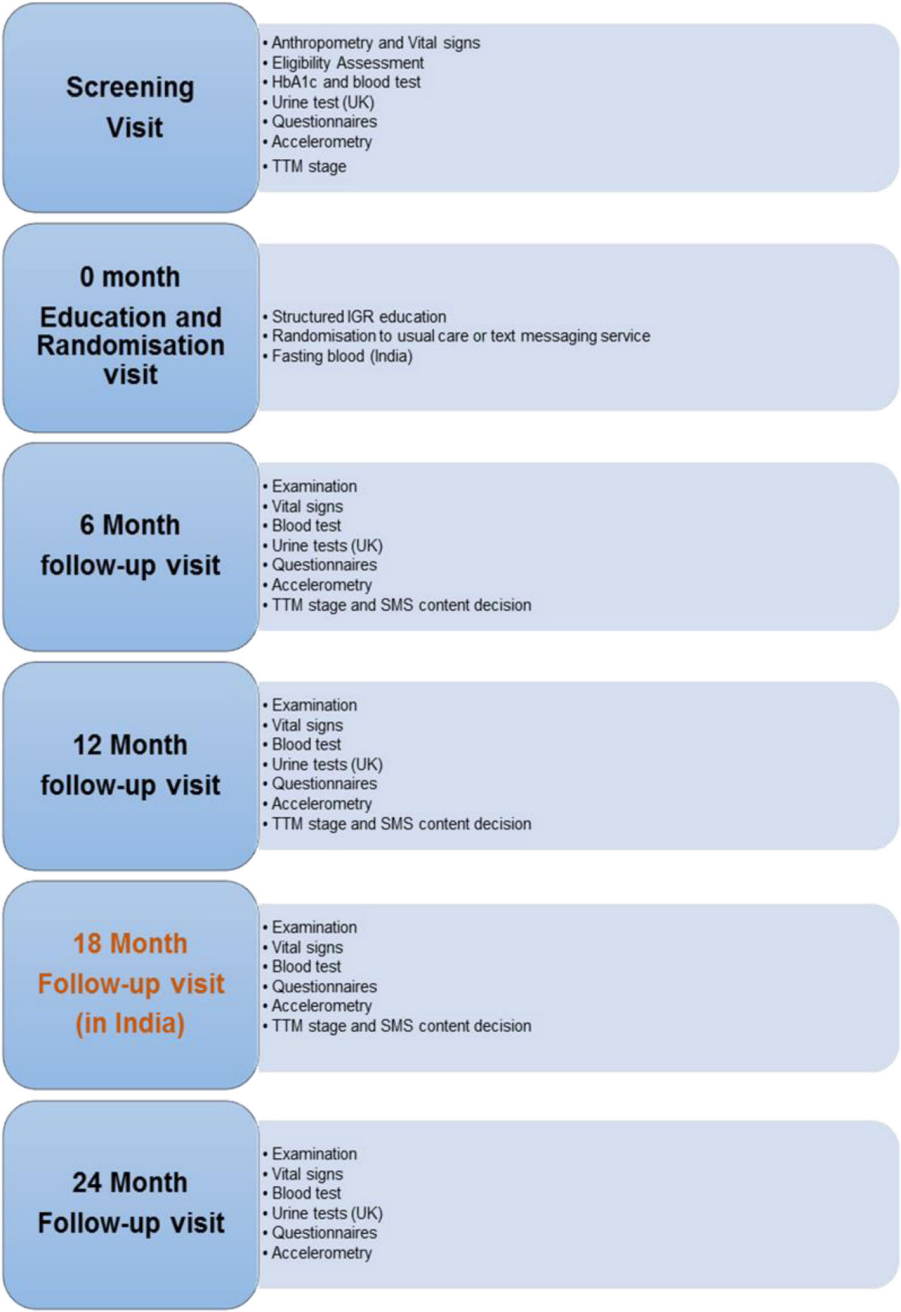
Clinical assessments and other activities at each visit

The questionnaires employed in the project have been validated or used previously in similar settings. The EQ-5D-3 L in India (the EQ-5D-5 L in the UK) is a validated quality of life questionnaire which measures the effects of disease and health status on perceived quality of life [20, 21]. It describes 5 health related quality of life dimensions (mobility, self-care, usual activities, pain/discomfort, and anxiety/depression) all of which can take one of 3 or 5 responses depending on the level of severity. The Recent Physical Activity Questionnaire (RPAQ) provides a validated estimate of physical activity, enabling physical activity energy expenditure (PAEE) and time spent in different intensity levels to be calculated [22, 23]. Physical activity is classified as home activities, activity at work, recreation, and transport. The Food Frequency Questionnaire (FFQ), employed in the UK sample, is a validated questionnaire for calculation of intake of dietary energy and of the major food constituents [24–27]; it has also been compared to 24-h dietary recall [28–30]. The 24-h dietary recall, employed in India, has been used previously by the Indian investigators in this setting [14]. It enumerates the food ingested from morning until bedtime and records dietary habits, food choices, food frequency and quality. The SMS acceptability questionnaire was developed for our feasibility study of SMS in diabetes prevention [14]. It assesses acceptability of text contents and message frequency. The TTM questionnaire is described below in the section on interventions. Clinical assessments and other activities at each visit are shown in Fig. 2.

### Primary outcome

In both countries, the primary outcome is progression to diabetes.

In *India,* progression to diabetes is defined at any follow-up visit by HbA1c ≥ 6.5% (48 mmol/mol) [16, 19].

In the *UK*, progression to diabetes is defined by any national and international criterion (Fasting plasma glucose (FPG) or HbA1c) at any visit, or in another care setting. The criteria for diagnosis of diabetes [16, 19, 31] are:

1. Symptoms and FPG ≥7.0 mmol/l or HbA1c ≥ 6.5%.
2. No symptoms but one of the following;
  FPG ≥7.0 mmol/l and HbA1c ≥ 6.5%; tests are repeated after 4 weeks and FPG ≥7.0 mmol/l and/or HbA1c ≥ 6.5%.
  FPG < 7.0 mmol/l and HbA1c ≥ 6.5%; repeat HbA1c after 4 weeks and if the second result is ≥6.5%
  FPG ≥7.0 mmol/l and HbA1c < 6.5%; repeat FPG after 4 weeks and if the second result is ≥7.0 mmol/l.

The reason for the difference in definitions is the different clinical settings in the two countries. In India, with the trial conducted in and around the workplace by dedicated trial staff, HbA1c was the single measure of glycaemia employed. In the UK, with recruitment in primary care and continued primary care involvement over the 2 years thereafter, other plasma glucose based criteria were also accepted as these were often the measures employed by the primary care staff.

### Secondary outcomes

- The proportion of participants achieving HbA1c ≤ 6.0%.
- FPG.
- Weight, BMI, waist circumference.
- Systolic and diastolic blood pressure.
- Total cholesterol, HDL cholesterol, LDL cholesterol, triglycerides.
- HOMA-IR, HOMA-β.
- Intakes of total energy, fat and carbohydrates.
- PAEE from self-report: total, home, work, commuting, leisure activity and total physical activity, time spent in moderate vigorous physical activity, time spent sedentary, total movement volume and intensity distribution (from accelerometry).
- Summary measure of quality of life from EQ-5D.
- Acceptability of SMS as a communication medium.

### Design/randomisation

Participants are randomised to the control or intervention group. In the intervention group, text messages are delivered 2–3 times weekly and contain educational, motivational and supportive content on diet, physical activity, and smoking. The message content varies depending on the participant’s TTM stage.

Lifestyle modification targets are in line with previously published diabetes prevention studies [32]:

1. Minimum 150 min moderate intensity exercise per week
2. A minimum of 7% weight loss or achieving a BMI of 25 kg/m^2^ (23 kg/m^2^ in India)
3. < 30% of total dietary energy intake from fat
4. < 10% of total dietary energy intake from saturated fat
5. > 15 g fibre per 1000 kcal dietary intake

Data are stored in a secure, non-identifiable manner for later statistical analysis.

Randomization to the intervention or control group is performed in *India* using a computer-generated randomization sequence, Matlab randperm version 6 based on Marsaglia’s algorithm [33]. In the *UK*, randomisation is provided by a commercial organisation (sealedenvelope.com) and performed in random permuted blocks with stratification by sex [male, female], age [< 35, 35–50, 50–65, > 65 years] and body mass index [< 28, 28–32, > 32 kg/m^2^].

### Setting

In *India*, pre-screening based on risk factors is performed in the workplace, having obtained prior permission from employers. Employees provide informed consent to undergo pre-screening, screening and trial entry and may withdraw at any time. In the *UK*, pre-screening occurs in primary care using the NHS Health Checks or other in-house schemes. If eligible, informed consent is obtained. Trial visits take place in CRN sites in NHS premises close to participants’ homes.

### Ethics, informed consent, and safety

The study is conducted in accordance with the recommendations for physicians involved in research on human subjects adopted by the 18th World Medical Assembly, Helsinki 1964 and later revisions.

Written consent is obtained from each participant after full explanation, an information leaflet offered and time allowed for consideration. The right of the participant to refuse to participate, or continue participating, without giving reasons and without prejudicing further treatment is respected. The Chief Investigator in each country ensures that participant confidentiality is respected and local data protection requirements are met.

In *India*, ethical approval is in place from an independent Institutional Review Board. In the *UK*, approval is from the Westminster Research Ethics Committee and Site Specific Assessment (SSA) plus R&D approvals were in place at each participating NHS Trust. Imperial College Academic Health Science Centre acts as the main Sponsor. Delegated responsibilities are assigned to the NHS trusts taking part in this study. Only research staff trained in Good Clinical Practice participate in the project to obtain informed consent and conduct procedures.

The study is subject to inspection and audit by the sponsors, ensuring adherence to Good Clinical Practice and other aspects of research governance.

### Eligibility – *Inclusion criteria*

HbA1c was chosen as the measure on which to define eligibility; it has the advantage over measuring circulating free glucose that it requires only random blood samples as opposed to fasting samples or samples timed in relation to ingestion of oral glucose. It reflects average circulating glucose levels over the preceding 4–8 weeks and has similar biological significance (in terms of the complications of diabetes) to that of free glucose measurements. Following standardisation by the International Federation of Clinical Chemistry, it is recognised internationally as a diagnostic test for diabetes with a cut-off at ≥6.5% [31]. For prediabetes, although the American Diabetes Association has recommended the HbA1c range of 5.7–6.4% [31], international consensus has not been reached on this. We are using 6.0–6.4% for recruitment in both India and the UK to include only patients at very high risk. In the UK, men and women with prediabetes aged 18 to 74 years and HbA1c levels in this range are recruited. The age range in India is 35 to 55 years.

### Eligibility – *Exclusion criteria*

#### Subjects are excluded if

HbA1c values render the participant eligible (HbA1c 6.0% to ≤6.4%) but FPG is in the WHO diagnostic range for diabetes (≥7.0 mmol/l) – a necessary requirement as people in the UK were assessed by any WHO criterion; pregnant or planning pregnancy; breastfeeding; enrolled in other clinical trials; active malignancy or under investigation for malignancy; unable to follow the protocol (Version 9, 17/03/2015) for any reason. FPG above the WHO diagnostic range was considered an exclusion criterion as it is a primary outcome in the UK.

#### Estimated timeline

Participants are followed up for 2 years.

#### Interventions

Usual care is given to both the groups and consists of a one-to-one interview, delivering personalised diet and exercise advice, supplemented by standard written material and education regarding prediabetes and diabetes. Usual care is delivered once at the beginning of the study.

Participants in the intervention arm receive tailored text messages based on healthy lifestyle principles. Messages are sent 2–3 times a week and tailored to the participants’ TTM stage.

A database of SMS text messages had been developed based on previous work [14], modified and expanded for use in both India and the UK. In addition, for the UK site, a Patient and Public Involvement Group in the CRN provided input into the SMS message design and content. The messages provide tips, suggestions, and positive reinforcement or encouragement for healthy behaviours. Content includes goal setting and self-monitoring, understanding intensity of physical activity, dietary energy and portion control, personal strategies for lifestyle change and overcoming barriers. Routine physical activity, time management, meal planning, and strategies for eating out also feature. To keep the text messaging novel and non-repetitive, the type and content of the messages change from day to day, and attempts are made to minimise message repetition.

Message content is modified depending on TTM stage. TTM is a behavioural theory which is widely applied in health research interventions aiming to induce behavioural change [34]. It is derived from key theories in psychotherapy. The four core constructs of the model are the “stages of change,” “processes of change,” “decisional balance,” and “self-efficacy”. The processes of change relate to ‘how’ individuals change their behaviour. They include cognitive, affective, evaluative and behavioural strategies that an individual may adopt to modify behaviour. The TTM is implemented in this study to assess an individual’s status in terms of behavioural change and to attempt to improve it. Individuals’ TTM stage is assessed at the start of the study and followed during the 2 year period.

### Safety assessments

The trial is conducted in compliance with Good Clinical Practice principles with which all investigators are confirmed to be familiar and the applicable regulatory requirement(s).

The study is subject to inspection and audit by Imperial College London under their remit as sponsor and other regulatory bodies to ensure adherence to Good Clinical Practice in Research and the NHS Research Governance Framework for Health and Social Care.

### Data

Data is locally collected and transferred to the central database. The data is coded and anonymised and then stored in an encrypted folder in a password protected computer.

### Statistical analysis

#### Sample size

Diabetes prevention trials that employ intensive lifestyle intervention and monitoring have achieved reductions in progression to diabetes in those at risk of between 36 and 65%. Based on earlier data from the Indian Diabetes Prevention studies, a 2 year conversion rate to diabetes of 25% could be expected in people with prediabetes in the absence of intervention. We have reported a 36% reduction in 2-year risk in a trial of SMS for diabetes prevention in 537 men in India [14]. In order to detect reductions in risk of 20% and 30% with 80% power at 5% significance, and allowing for 4% dropout, we estimated that recruitment totals of 2268 and 976 respectively, would be required. We therefore chose 20% as a nominal expectation for the minimum risk reduction, requiring a sample of 2268 participants, 1134 in each of the two groups to be compared.

#### Data reduction

The raw 50 Hz triaxial accelerometry data were converted into vector magnitude (VM) activity counts in 5-s epochs. We classified the intensity distribution in fine-grained bins, and also collapsed into broader intensity categories of sedentary time (< 200 counts per min), light (200–2500 cpm), moderate-to-vigorous PA (> 2500 cpm); the latter level discriminates between slow walking and higher intensity locomotion [30]. Non-wear time was defined as prolonged (> 90 min) periods of inactivity; this was taken into account using a cosine method [35] when summarising total volume (average intensity) and time spent in different intensity categories for each participant and visit. Individual records with less than 72 h of wear time and less than 8 h wear in each time quadrant of the day were excluded from analysis.

#### Analysis plan

Analyses will be performed using Stata version 14.2 [36] and R [37] and will follow the recommendations of the CONSORT 2010 statement (www.consort-statement.org).

The primary efficacy outcome will be development of Type 2 diabetes, defined as HbA1c ≥ 6.5% in India, and any WHO criterion in the UK.

A hazard ratio comparing the intervention vs control group for Type 2 diabetes, together with a 95% confidence interval and *p*-value will be estimated using a discrete-time proportional hazards model [38], which takes into account the fact that the data are interval censored, i.e. HbA1c is measured at protocol-defined visits [6,12,(18), 24 months], so in individuals who develop diabetes (HbA1c ≥ 6.5%), the exact time that it developed is not known. Individuals who withdraw from the study or who have not developed Type 2 diabetes by the end of follow-up will be censored at their last available follow-up time.

For each of the continuous secondary efficacy outcomes measured at multiple time points during follow-up, the mean and standard deviation of the values at each time point will be calculated. Differences between the intervention and control groups at each time point and 95% CI will be estimated from a linear regression model with random intercepts at the individual level (to allow for repeated measures), including the baseline value of the outcome and parameters for randomized group, time and randomized group-by-time interaction. For the objectively measured physical activity outcomes, accelerometer wear time will also be included in the model. Outcome variables with a skewed distribution (Triglyceride, HOMA-IR, HOMA-β) will be summarized using the median and Interquartile Range and log transformed before being analysed using the model described above.

All outcomes will be analysed including all individuals in the group to which they were randomized, regardless of whether the intervention was actually received (based on the Intention-to-Treat principle). The primary efficacy outcome will also be analysed using a Per Protocol population, which reclassifies individuals in whom the intervention was not deemed to have been successfully delivered because text messages had been blocked on the mobile phone during the first 6 months of follow-up.

The analyses described above will be performed separately on data from India and the UK, and also on a combined dataset, where models will be adjusted for country. For the primary efficacy outcome, the interaction between randomised group and (1) country, (2) sex will be tested by including the relevant multiplicative interaction parameter in the model. Estimated hazard ratios for type 2 diabetes and 95% confidence intervals will be reported separately in men and women.

### Publication policy

The study results will be disseminated by peer reviewed scientific journals, internal report, conference presentation and publication on websites. No identifiable personal data will be published. All anthropometry and personal clinical data will be expressed as mean/ median and spread of the population in the study. All participants will be informed of the results at the conclusion of the study and details of any publications that arise from the study will be disseminated to participants.

### Sponsor

The sponsor for the study is Joint Research Compliance Office, Imperial College London and Imperial College Healthcare NHS Trust.

## Discussion

The study aims to discover whether progression from prediabetes to type 2 diabetes can be reduced with tailored text messaging (in addition to standard lifestyle advice) in two different environments (India and the UK). If the result is positive, text messaging could provide an inexpensive adjunct to diabetes prevention programmes globally.

The clinical settings in India and the UK are very different, necessitating different recruitment strategies in the two countries (workplace in India and primary care in the UK), criteria for pre-screening and criteria for diagnosis (although in both countries the criteria are compatible with national and international recommendations [17, 18]). In India the study is conducted with employers’ consent close to the workplace by dedicated staff whereas in the UK, NIHR CRN staff performed most of the studies as part of their wider clinical research remit. As a result, and for pragmatic reasons the UK site omits the 18-month visit. The text message content is also slightly modified in each country to ensure compatibility with the dietary and physical activity behaviours in the two settings. Within one country, the same intervention and assessment methodologies are applied to all participants.

## Abbreviations

BMI: Body mass index
cpm: Counts per min
CVD: Cardiovascular disease
FFQ: Food Frequency Questionnaire
FPG: Fasting plasma glucose
HbA1c: Haemoglobin A1c
NHS: National Health Service
NIHR CRN: National Institute for Health Research Clinical Research Network
PAEE: Physical activity energy expenditure
RPAQ: Recent Physical Activity Questionnaire
SMS: Short messaging service
TTM: Transtheoretical Model of Behavioural Change
WHO: World Health Organisation
